# Stability of beta-titanium T-loop springs preactivated by gradual curvature

**DOI:** 10.1590/2177-6709.22.6.061-067.oar

**Published:** 2017

**Authors:** Sergei Godeiro Fernandes Rabelo Caldas, Renato Parsekian Martins, Marcela Emílio de Araújo, Marília Regalado Galvão, Roberto Soares da Silva, Lídia Parsekian Martins

**Affiliations:** 1 Universidade Federal do Rio Grande do Norte, Departamento de Odontologia (Natal/RN, Brazil).; 2 Universidade Estadual Paulista, Faculdade de Odontologia de Araraquara, Programa de Pós-graduação em Ciências Odontológicas - Ortodontia (Araraquara/SP, Brazil).; 3 Private practice (Araraquara/SP, Brazil).; 4 Universidade Estadual Paulista, Faculdade de Odontologia de Araraquara, Departamento de Clínica Infantil (Araraquara/SP, Brazil).

**Keywords:** Orthodontics, Tooth movement, Orthodontic wires

## Abstract

**Objective:**

Evaluate changes in the force system of T-Loop Springs (TLS) preactivated by curvature, due to stress relaxation.

**Methods:**

Ninety TLSs measuring 6 x 10 mm, produced out with 0.017 x 0.025-in TMA^®^ wire and preactived by gradual curvature, were randomly distributed into nine groups according to time point of evaluation. Group 1 was tested immediately after spring preactivation and stress relief, by trial activation. The other eight groups were tested after 24, 48 and 72 hours, 1, 2, 4, 8 and 12 weeks, respectively. Using a moment transducer coupled to a digital extensometer indicator adapted to a universal testing machine, the amount of horizontal force, moment and moment-to-force ratios were recorded at every 0.5 mm of deactivation from 5 mm of the initial activation, in an interbracket distance of 23 mm.

**Results:**

The horizontal forces decreased gradually among the groups (*p*< 0.001) and the moments showed a significant and slow decrease over time among the groups (*p*< 0.001). All groups produced similar M/F ratios (*p*= 0.532), with no influence of time.

**Conclusions:**

The TLSs preactivated by curvature suffered a gradual deformation over time, which affected the force system, specifically the moments, which affected the horizontal forces produced.

## RESUMO

**Objetivo:**

avaliar as mudanças no sistema de forças das molas T pré-ativadas por curvatura, devido ao alívio de tensão estrutural.

**Métodos:**

noventa molas em forma de “T”, medindo 6 mm x 10 mm, confeccionadas com fio de beta-titânio TMA^®^ de 0,017” x 0,025” e pré-ativadas por curvatura gradual, foram distribuídas aleatoriamente em nove grupos, de acordo com o momento de avaliação. O Grupo 1 foi testado imediatamente após a pré-ativação da mola e liberação do estresse, por meio de simulação da ativação. Os outros oito grupos foram divididos conforme o intervalo de tempo testado: após 24, 48 e 72 horas; 1, 2, 4, 8 e 12 semanas. Usando um transdutor de momentos acoplado a um indicador digital para extensometria e adaptado a uma máquina universal de ensaios, as magnitudes da força horizontal, do momento e da proporção momento/força (M/F) foram registradas a cada 0,5 mm de desativação a partir da ativação inicial de 5 mm, utilizando-se uma distância interbraquetes de 23 mm.

**Resultados:**

as forças horizontais reduziram-se gradualmente nos grupos (*p*< 0,001) e o momento apresentou uma queda lenta e significativa ao longo do tempo (*p*< 0,001). Todos os grupos tiveram proporções M/F semelhantes (*p*= 0,532), não influenciadas pelo período de tempo.

**Conclusões:**

as molas pré-ativadas por curvatura gradual sofreram deformação progressiva ao longo do tempo, o que afetou o sistema de forças - especificamente o momento -, alterando as forças horizontais produzidas.

## INTRODUCTION

The β-Ti T-loop spring (TLS) has been used since the 80’s for space closure due to its alloy and design advantages.^1^ ^,^ ^2^ Even though the specific design produces a high moment of activation, this moment might not be enough to move teeth by translation. Thus, the addition of a residual moment is needed by increasing the angulation of the extremities of the loops, in a procedure known as preactivation.^3^ Templates^4^ and other methods^5^ of preactivation have been developed to produce specific forces and enough moment-to-force (M/F) ratios, allowing different types of tooth movement.

**Figure 1:**
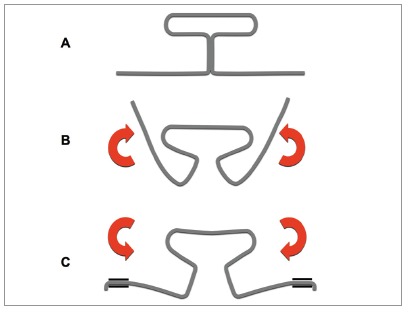
A) TLS in passive form. B) TLS preactivated by gradual curvature. C) TLS engaged to brackets (loaded in an opposite direction to the preactivation).

Upon engagement to the brackets, loops are normally loaded in the opposite direction of the preactivation, which could over time cause progressive deformation and force reduction (Fig 1).^6^ ^,^ ^7^ This time-dependent effect is called stress relaxation and has been thoroughly studied in the alloys used in Orthodontics.^8^ ^-^ ^13^ Besides the fact that this effect has only been superficially evaluated for β-Ti,^9^ ^,^ ^11^ ^,^ ^13^ ^-^ ^15^ it has never been studied on TTLs preactivated by gradual curvatures, which might be less sensitive to stress relaxation. 

Thus, the aim of this study was to evaluate changes in the force system of TLSs preactivated by curvature due to stress relaxation.

## MATERIAL AND METHODS

**Figure 2:**
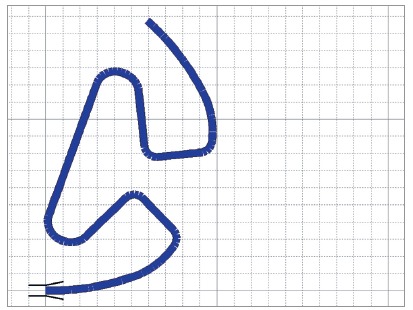
Template developed in the Loop software (dHAL Orthodontic Software, Athens, Greece) used to preactivate the TLS. The software allows the template to be printed in 1:1 ratio (each square measures 1 mm^2^).

Ninety TLSs measuring 6 x 10 mm were bent with a “Marcotte” plier (Hu-Friedy Dental Instruments, Chicago, USA) using 0.017 x 0.025-in β-Ti (TMA^®^, Ormco Corporation, Glendora, USA) wires and preactivated by gradual curvature following a template (Fig 2).

The choice of symmetrical springs was made due to a specification of the test system used, as it only allows measurements of this type of spring, since the horizontal force is measured at the top of the device and the moment at the bottom. Asymmetric springs generate different forces and moments in both ends. In addition, the occurrence of a geometry change alters the force system.

**Figure 3:**
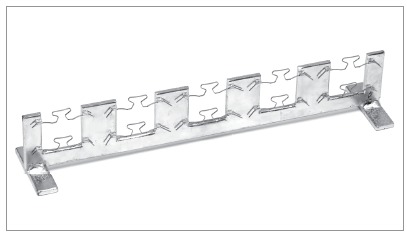
Custom-made device in order to keep the TLSs activated 5 mm.

The TLSs were randomly divided into nine groups according to the time of evaluation. Group 1 was tested immediately after preactivation and stress relief, by trial activation. The other eight groups were maintained at 5 mm activation for different times in an interbracket distance (IBD) of 23 mm, after the same procedures were made to them. A custom-made device was specifically made to keep the TLSs in position on a similar way that they would remain clinically (Fig 3). Groups 2, 3, 4, 5, 6, 7, 8 and 9 were kept restrained for 24, 48 and 72 hours; 1, 2, 4, 8 and 12 weeks, respectively.

An universal testing machine (EMIC, São José dos Pinhais, Brazil), equipped with a load cell of 0.1 kN, was coupled to a moment transducer and a digital extensometer indicator (Transdutec, São Paulo, Brazil) for the tests. The speed used for the test was 5 mm/min and the digital extensomer’s excitation and sensitivity was 5V and 0.5 mV/V, respectively.^14,16,17^ For the test, the TLSs were positioned symmetrically in an IBD of 23 mm. 

**Figure 4:**
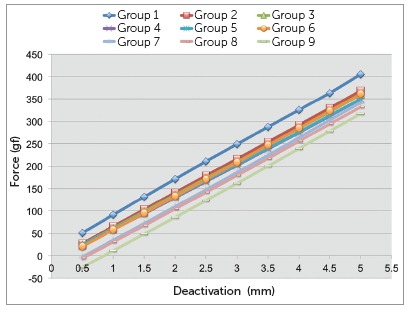
Chart depicting the average forces produced during deactivation from 5 to 0.5 mm for the groups tested.

Horizontal force and moments were recorded for every 0.5 mm of deactivation after 5mm of initial activation, and M/F ratios were calculated. The amount of horizontal overlap of the vertical extensions of the TLSs in “neutral position” was also calculated by linear interpolation. The load/deflection (L/D) ratio (the slope of the deactivation graph) was also obtained based in the graph (Fig 4).

SPSS v. 16.0 (SPSS Inc., Chicago, USA) statistical analysis software was used in this study. The Kolmogorov-Smirnov test indicated normal distributions and Levene’s test showed that all variables had similar variances, except the M/F ratios.

The multivariate profile analysis using the procedure for analysis of repeated measures was used in order to detect differences of forces, moments and M/F ratios among the groups. This analysis compares the total profile, or deactivation pattern, of a whole group in relation to time and deactivation. In order to identify the differences among the groups, the post-hoc Tukey test was used with the averages generated by each time (total profiles average).

ANOVA was used, at level of 5%, to detect differences among the groups on L/D ratio and the amount of overlapping of the vertical extensions of the TLSs in “neutral position”. Post-hoc Tukey test, at level of 5%, was used to identify the groups’ differences.

## RESULTS

There was a significant decrease of force over time among the groups when the total profiles of the TLSs were compared (*p*< 0.001) (Table 1). The total profiles horizontal forces decreased gradually among the groups (Table 2 and Fig 4) with values of 228.4 gf, 197.3 gf, 191.4 gf, 184.5 gf, 185.9 gf, 189.9 gf, 167.8 gf, 161.4 gf and 143.7 gf for groups 1 through 9. Also, there was significant interaction of the time on the rate of force decrease of the loops (*p*= 0.006) among the groups (Table 1), meaning that the load-deflection rate of the loops also decreased with time. The values (Table 3) ranged from 78.1 to 72.1 gf per 0.5 mm and did not obey a linear decrease.

**Table 1:** Multivariate profiles test significance for force (F), moment (M) and M/F variables.

Variable	Force	Moment	M/F
	*p value*	*p value*	*p value*
Time	< 0.001	< 0.001	0.532
Deactivation	< 0.001	< 0.001	0.124
Deactivation x Time	0.006	0.048	0.159

**Table 2:** General profiles means and standard deviations for forces and moments within groups.

Group	Force			Moment		M/F
	Mean	SD	Mean	SD	Mean	SD
Group 1	228.4 A	115.1	1941.0 A	272.9	12.7	13.3
Group 2	197.3 AB	112.8	1919.9 A	336.8	-5.8	180.1
Group 3	191.4 AB	110.3	1922.0 A	301.9	17.3	42.3
Group 4	184.5 ^B^	107.4	1912.9 A	317.5	3.7	100.2
Group 5	185.9 ^B^	109.7	1840.7 A	286	20.9	61.7
Group 6	189.9 ^B^	110.9	1775.4 A	288.9	24.8	90.8
Group 7	167.8 ^BC^	112.9	1713.3 AB	286.7	13.3	40.2
Group 8	161.4 ^BC^	110.1	1506.8 ^B^	287.8	-3.5	245.3
Group 9	143.7 ^C^	112.2	1486.4 ^B^	256	18.8	49

Different superscript letters indicate group differences.

**Table 3:** Means and standard deviations for the overlapping of the vertical extensions of the TLSs and L/D ratio.

Group	Neutral Position* (mm)		L/D** (gf/0.5mm)^#^	
	Mean	SD	Mean	SD
Group 1	-0.18 A	0.31	78.1 A	3.8
Group 2	0.11 AB	0.53	75.5 AB	4.4
Group 3	0.18 AB	0.29	74.3 AB	3.4
Group 4	0.19 AB	0.34	72.2 ^B^	3.6
Group 5	0.17 AB	0.45	72.1 ^B^	3.2
Group 6	0.23 AB	0.26	75.5 AB	3.4
Group 7	0.54 ^BC^	0.32	76.1 AB	3.0
Group 8	0.59 ^BC^	0.22	74.7 AB	2.9
Group 9	0.85 ^C^	0.29	75.7 AB	3.9

*p < 0.001. **p = 0.008. ^#^ in order to acquire the L/D per mm, multiply the values times 2. Different superscript letters indicate group differences.

The amount of overlap of the vertical extensions of the TLSs (addressed as “neutral position” on Table 3) decreased gradually from group 1 (-0.18 mm) to group 9 (0,85 mm) (*p*< 0.001). 

**Figure 5:**
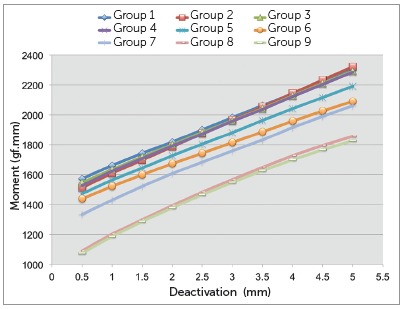
Chart depicting the average moments produced during deactivation from 5 to 0.5 mm for the groups tested.

There was a significant and slow decrease of moment over time among the groups when the total profiles of the TLSs were compared (*p*< 0.001) (Table 1). The total profile average moments produced throughout the deactivation were similar among group 1 (1941.0 gf.mm) to 7 (1713.3 gf.mm), being different only from group 8 (1506.8 gf.mm), which was similar to group 7 (1713.3 gf.mm), and group 9 (1486.4 gf.mm) (Table 2). There was also a significant interaction between evaluation time and deactivation (*p*< 0.048) (Table 1 and 2, Fig 5).

**Figure 6:**
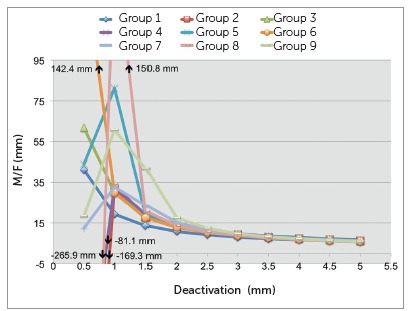
Chart depicting the average M/F ratios produced during deactivation from 5 to 0.5 mm for the groups tested.

All groups produced similar total profile average M/F ratios (*p*= 0.532): 12.7 mm, -5.8 mm, 17.3 mm, 3.7 mm, 20.9 mm, 24.8 mm, 13.3 mm, -3.5 mm and 18.8 mm, for groups 1 through 9, respectively. No interaction was found between time evaluation and deactivation (Table 1 and 2, Fig 6). 

## DISCUSSION

**Figure 7:**
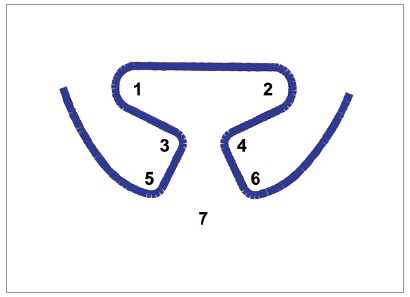
TLSs structural angles measured for finding the deformation location. The angle 7 is formed by the intersection of horizontal extremities of the TLS.

There was a gradual force decrease among the groups over time, critical in groups 4 (72 hours) and 9 (12 weeks), which can be explained by the stress relaxation phenomenon. This decrease of force over time is in agreement to several other reports that have measured this effect on straight wires, showing it to be time-dependent.^9,11,13^ There were only two studies^14^ ^,^ ^18^ that looked upon this effect in more elaborate configurations, evaluating stress relaxation on TLSs preactivated by bends, in which a decrease of 15.5% on the force levels of TLSs was shown in the first 24 hours. The present results also show a decrease over time, but with a different behavior that is probably due to the gradual curvature that was used for preactivation, distributing stress over the entire extent of curvature. The structural area of the TLS that was affected and responsible for relaxation was the angle between the vertical and horizontal extremities of the spring, which is a concentrated bend. This was found by scanning the TLSs of groups 1, 4 and 9 immediately before testing and measuring the TLS’s structural angles with the Screen Protractor 4.0 software (Iconico, New York, USA). Method error assessed through intraclass correlation coefficient (ICC) showed high reliability (0.996) (Table 4 and Fig 7).

**Table 4:** ANOVA and post-hoc Tukey test to identify which angle (according to Fig 7) suffers a deformation due to time.

Angle (Fig. 7)	Neutral Position Group 1		Post Period Group 4		Post Period Group 9		p
	Mean	SD	Mean	SD	Mean	SD	
1	20.65	2.42	20.85	2.28	20.60	2.66	0.097
2	20.21	2.01	21.65	2.82	23.13	2.95	0.063
3	92.10	2.98	92.34	2.55	92.69	1.48	0.864
4	92.60	2.26	94.38	3.10	94.32	2.16	0.226
5	60.37	4.39 A	74.74	4.88 ^B^	73.11	8.29 ^B^	<0.001
6	64.66	3.88 A	71.47	4.20 ^B^	79.46	5.48 ^C^	<0.001
7	87.00	5.30 A	107.85	14.93 ^B^	113.24	12.26 ^B^	<0.001

Different superscript letters indicate group differences.

Time had a significant effect on L/D ratio, which was not constant throughout the evaluation, as demonstrated by the interaction between time and deactivation (force variation or L/D ratio) (Table 1). This effect, although significant, shows very small differences (6 gf/0.5mm) (Table 3), which may be clinically insignificant. Since the shape of a device can influence L/D ratio,^19^ this differences could have happened due to shape differences in the TLS, as different places in its structure go through stress relaxation, causing slightly different L/D ratios overtime. 

Moments were also affected by time, however, there was a significant decrease on the moments produced by the TLSs over time only in groups 8 and 9. It is known that an increase in the intensity of preactivation increases the moments produced, so a decrease in the preactivation due to stress relaxation of the gable could explain a decrease in the moment values.^3^ ^,^ ^20^ ^-^ ^23^ In the present results, the behavior of overactivation caused by “neutral position”, due to the overlap of the vertical extensions of the TLSs, decreased over time and was consistent to the gradual decrease on the horizontal forces, since it has an effect on it. The clinician should be aware that when these changes occur in the shape of the TLS, this can decrease the horizontal force produced due to a reduction of the overlap of vertical extensions,^24^ and therefore this effect should be compensated.^14^

Regarding the horizontal force, there was a significant interaction of time on the rate of variation of the moments with deactivation (*p*< 0.048). With time, smaller decreases of moments are expected, since some relaxation of the TLS already took place. The moment levels during deactivation on the first four weeks can be fit on a relatively straight line, which over time, turns out to be a slight curvature (Fig 5). This effect has already been shown on the literature and is explained by deformations in specific parts of the loop.^14^

The M/F ratios did not change and there was not interaction of time on the rate of the M/F ratios levels with deactivation. This was probably due to the fact that the stress relaxation in the wire happened slowly on moments, causing a gradual decrease on the overlapping of the vertical extensions of the TLSs, which proportionally decreased the force levels. 

Due to the laboratory characteristics of this study, the extrapolation of data to the clinical routine should be performed with caution. From the obtained results, it is suggested that the force system is more stable in the absence of concentrated bends, recommending that orthodontists should activate these devices by gradual curvatures.

## CONCLUSIONS

The T-loop springs preactivated by curvature suffered a gradual deformation over time, which affected the force system, specifically the moments, which affected the horizontal forces produced. 

Even though a gradual curvature distributes stress over the wire, the structure of the T-loop springs relaxes in an area of sharp bend, inherent to the “T” shape.
